# Peer Review of “Effects of Interventions for the Prevention and Management of Maternal Anemia in the Advent of the COVID-19 Pandemic: Systematic Review and Meta-Analysis”

**DOI:** 10.2196/82836

**Published:** 2025-10-06

**Authors:** Anonymous

**Keywords:** maternal anemia, anemia in pregnancy, COVID-19, pregnancy complications, meta-analysis, maternal and child health, anemia prevention, reproductive health

*This is the peer-review report for “Effects of Interventions for the Prevention and Management of Maternal Anemia in the Advent of the COVID-19 Pandemic: Systematic Review and Meta-Analysis.”*

## Round 1 Review

Some weaknesses, gaps, and limitations in this study [1] based on the current study provided could include:

1. Retrospective studies: The majority of the studies included in the meta-analysis were retrospective epidemiological studies, which may have limitations in terms of bias, data accuracy, and causality compared to prospective studies or randomized controlled trials. This could affect the reliability and generalizability of the findings.
2. Heterogeneity: The high heterogeneity identified in the pooled effect estimates suggests variability in study designs, interventions, and outcomes across the included studies. This heterogeneity can impact the interpretation of the results and the ability to draw consistent conclusions.
3. Publication bias: The presence of publication bias indicated by the asymmetrical funnel plot could introduce bias in the pooled effect estimates. This bias may be due to the selective reporting of studies with significant results, potentially skewing the overall findings.
4. Limited scope: Some studies may not have clearly defined the age range of participants or the specific stage of the gestation period analyzed. A lack of detailed information on these aspects could limit the applicability and generalizability of the results to specific subgroups of pregnant women.
5. Indirect effects of COVID-19: While the study focused on the direct impact of COVID-19 on maternal anemia interventions, indirect contributions of the pandemic on anemic conditions may not have been fully elucidated. Understanding these indirect effects could provide a more comprehensive view of the challenges faced during the pandemic.
6. Effectiveness trends: The decreasing trend in the effectiveness of interventions against maternal anemia from 2020 to 2022 raises questions about the sustainability and adaptability of intervention strategies, especially in the context of global health emergencies. Further research is needed to explore the reasons behind this trend and potential strategies for improvement.

Addressing these weaknesses and limitations could enhance the validity and applicability of the study findings and contribute to a more comprehensive understanding of the impact of COVID-19 on maternal anemia interventions.

Overall, the meta-analysis highlighted the challenges faced in addressing maternal anemia during the COVID-19 pandemic and the need for continuous monitoring and adaptation of intervention strategies to mitigate adverse outcomes.
